# Low Overpotential Electrochemical Reduction of CO_2_ to Ethanol Enabled by Cu/Cu_x_O Nanoparticles Embedded in Nitrogen-Doped Carbon Cuboids

**DOI:** 10.3390/nano13020230

**Published:** 2023-01-04

**Authors:** Monther Q. Alkoshab, Eleni Thomou, Ismail Abdulazeez, Munzir H. Suliman, Konstantinos Spyrou, Wissam Iali, Khalid Alhooshani, Turki N. Baroud

**Affiliations:** 1 Department of Mechanical Engineering, King Fahd University of Petroleum & Minerals, Dhahran 31261, Saudi Arabia; 2 Department of Materials Science and Engineering, University of Ioannina, 45110 Ioannina, Greece; 3 Interdisciplinary Research Center for Membranes and Water Security, King Fahd University of Petroleum & Minerals, Dhahran 31261, Saudi Arabia; 4 Interdisciplinary Research Center for Hydrogen and Energy Storage (IRC-HES), King Fahd University of Petroleum & Minerals (KFUPM), Dhahran 31261, Saudi Arabia; 5 Department of Chemistry, King Fahd University of Petroleum & Minerals, Dhahran 31261, Saudi Arabia; 6 Interdisciplinary Research Center for Refining and Advanced Chemicals, King Fahd University of Petroleum and & Minerals, Dhahran 31261, Saudi Arabia; 7 Department of Materials Science and Engineering, King Fahd University of Petroleum & Minerals, Dhahran 31261, Saudi Arabia

**Keywords:** electrochemical reduction, CO_2_, ethanol, low overpotential, porous carbon support, Cu nanoparticles

## Abstract

The electrochemical conversion of CO_2_ into value-added chemicals is a promising approach for addressing environmental and energy supply problems. In this study, electrochemical CO_2_ catalysis to ethanol is achieved using incorporated Cu/Cu_x_O nanoparticles into nitrogenous porous carbon cuboids. Pyrolysis of the coordinated Cu cations with nitrogen heterocycles allowed Cu nanoparticles to detach from the coordination complex but remain dispersed throughout the porous carbon cuboids. The heterogeneous composite Cu/Cu_x_O-PCC-0h electrocatalyst reduced CO_2_ to ethanol at low overpotential in 0.5 M KHCO_3_, exhibiting maximum ethanol faradaic efficiency of 50% at −0.5 V vs. reversible hydrogen electrode. Such electrochemical performance can be ascribed to the synergy between pyridinic nitrogen species, Cu/Cu_x_O nanoparticles, and porous carbon morphology, together providing efficient CO_2_ diffusion, activation, and intermediates stabilization. This was supported by the notably high electrochemically active surface area, rich porosity, and efficient charge transfer properties.

## 1. Introduction

Compared to preindustrial times, the average global temperature has increased by an amount of 1.0 °C as per the latest reports [1,2]. CO_2_ is believed to be the major contributor to this issue, reaching an unprecedented concentration of over 410 ppm in 2020 [3]. As such, CO_2_ mitigation has become a research focus in the recent past. Direct electrochemical conversion of CO_2_ is one of the most promising solutions for mitigating CO_2_ being compatible with green energy technologies while being operated at ambient conditions [4,5]. Efforts are currently being devoted to improving reaction selectivity towards hydrocarbons and alcohols to address the global energy problem as well as the environmental crisis. However, such products develop increasingly complex mechanisms and involve multi-electron reductions of different intermediates [6].

Early and modern research on CO_2_ electrochemical reduction reliably established the dependence of conversion products on the cathode materials, having copper as the only metal electrode capable of reducing CO_2_ into chemicals with more than 2e^−^ transfer at substantial quantities [7]. Among these products are several hydrocarbons, alcohols, and aldehydes. CO, HCOO^−^, C_2_H_4_, and CH_4_ have been identified as the major CO_2_ reduction products in aqueous KHCO_3_ electrolytes. While methane and ethylene showed 43% and 23% maximum FEs, respectively, alcohols have been reported in lower quantities [8,9,10]. Although relatively high product yields were achieved on the copper electrodes, the reported overpotentials were as high as 1 V [11]. Additionally, the deactivation of copper electrodes made them unsuitable for long-term use [12].

To improve C_2_ products selectivity at lower overpotentials, copper has been modified to take on different forms and structures. Electrodeposited Cu I oxide films [13], monodispersed Cu nanoparticles (NPs) [14], amorphous Cu NPs [15], Cu nanosheets [16], and nanoporous ribbon arrays [17] are examples of different Cu structures that generated C_2_ products. Those studies identified mass transport limitations of CO_2_, low surface areas, and particle aggregation as key factors for lowering the selectivity towards C_2_ products. Additionally, the local pH, the material’s uncoordinated sites, and surface defects play a significant role in the activation of CO_2_ [13,14,15,16,17].

Optimizing the above-mentioned parameters towards higher CO_2_ reactivity was made possible by incorporating Cu NPs into suitable support materials [11,18]. Carbonaceous support materials are known to have high electrical conductivity, stability, abundance, large surface area, and low cost [11]. Different types of carbons, such as carbon black [19], graphite sheets [20], reduced graphene oxide [21], CNTs [19,22], and porous carbon [23], have been reported as supports. Furthermore, nitrogen species can be introduced to the carbon structure to enhance the support’s electronic properties and activate the CO_2_ reduction reaction [24]. Up till now, several studies reported the doping of nitrogen into the different types of carbons such as N-doped CNT [25], MWCNT with pyrolyzed carbon nitride [26], N-doped carbon nanosheets [27], N-doped graphene quantum dots [28], and 3D graphene foam with nitrogen defects [29].

In electrocatalysis, doping and introducing defects are regarded as a successful strategy to improve catalysts activities [30,31,32,33,34]. In particular, nitrogen-modified porous carbon structures can function effectively as unique support materials. The porosity can significantly facilitate continuous reactions of intermediates, assist CO_2_ transport, and consequently enhance the electrochemically active surface area. Previously, embedded Cu nanoparticles into the MOF-derived (Metal–Organic Framework-derived) catalyst demonstrated successful CO_2_ reduction into C_2_ products. A total of 70.52% ethanol FE at −0.87 V vs. RHE was achieved on Cu particles loaded on a 3D geometric porous structure [35]. Such high activity was ascribed to the matrix connectivity of the carbon support. Oxide-derived Cu particles supported on MOFs also showed superb ethanol product selectivity at low overpotentials as the 3D porous structure minimized the diffusion resistance for mass transport. Along with the higher Cu_2_O content and optimum particle size, 35% FE to ethanol at −0.5 V vs. RHE was obtained [36]. Notably, C_2_ product selectivity can be influenced by the percentage of the pyridinic species in the nitrogen carbon copper system. Cu species supported on N-C microporous structure promoted C_2_ selectivity compared to carbon black support [37]. Monodispersed copper catalyst on a nitrogen-doped framework also displayed improved C_2_ product selectivity, which was attributed to the high dispersion of Cu particles and the facilitated adsorption of CO_2_ on a microporous framework [38]. C_2+_ products selectivity of 77% was reported in [39] through highly modular tricomponent complymer modified Cu electrodes. This performance was attributed to the increased local CO_2_ concentration, enhanced gas diffusion due to increased porosity, and the effect of local electric field [39]

Herein, porous carbon cuboids (PCC) serve as a support material for Cu/Cu_x_O nanoparticles synthesized through ultrafast coordination of 4,4′-bipyridine with copper cations. Under high carbonization temperatures, nitrogen atoms stack to Cu/Cu_x_O nanoparticles. The embedded Cu/Cu_x_O nanoparticles acted as active components facilitating the CO_2_ reduction reaction; the nitrogen heterocycles are also thought to further assist the reaction. In this study, the amount of Cu/Cu_x_O nanoparticles embedded in the carbon-nitrogen network was altered by varying the leaching time with a strong acid. Leaching the cuboids for 1 h resulted in 1.93 at% copper and leaching for 6 h resulted in 0.86 at%, while the unleached cuboids showed 16.93 at% Cu content determined from comparing copper to carbon peaks in XPS spectra. As such, the three composites were labeled Cu/Cu_x_O-PCC-0h, Cu/Cu_x_O-PCC-1h, and Cu/Cu_x_O-PCC-6h (where the number designates the leaching time in hours). The leaching effect was studied against CO_2_ reactivity and was shown to significantly increase the materials’ surface area, enrich the porosity, alter the nitrogen nature, and deplete the Cu nanoparticles. The effect of those parameters was reflected in the materials’ electrochemical performances. Results show that enhancing the materials’ porosity and surface area cannot have a positive impact without possessing an adequate number of active sites. In addition, pyridinic nitrogen seemed to be correlated to improved electrocatalytic performance.

## 2. Materials and Methods

### 2.1. Materials

4,4′-bipyridine (C_10_H_8_N_2_, 98%, Alfa Aesar, Haverhill, MA, USA), copper (II) chloride dihydrate (CuCl_2_·2H_2_O, ≥ 99%, Sigma-Aldrich, St. Louis, MO, USA), triblock copolymer Pluronic F127 (Sigma-Aldrich), nitric acid (HNO_3_, >65%, Boom BV, Meppel, The Netherlands), nafion solution 5 wt. % (Sigma-Aldrich) and ethanol (absolute) were all used as purchased without any further purification. Carbon paper was used as working electrode support. Nafion membrane 117 (Fuel Cell Store, College Station, TX, USA) was used as ion exchange membrane in the electrochemical cell. Potassium bicarbonate (99.97%, KHCO_3_, Sigma-Aldrich) was used for preparing electrolytes with high-purity deionized water.

### 2.2. Material Synthesis

The carbon cuboids were synthesized as per the previously reported procedure [40,41]. Briefly, a 100 mL solution of 0.1 M 4,4′-bipyridine water–ethanol was prepared with a 1:17 H_2_O: EtOH volume ratio. 1.0 g of the triblock copolymer (F127) was dissolved into the solution, denoted solution A. 900 mL of 5.6 mM CuCl_2_·2H_2_O aqueous solution was prepared, denoted solution B. Solution A was rapidly mixed with solution B, to ensure a fast reaction. The presence of F127 ensures the dispersity of the formed polymer colloids. During the reaction, 4,4′-bipyridine heterocycles bridge between Cu centers by pairing 2 N electrons to a Cu cation, forming a coordinated network complex. The products were formed immediately after mixing, were collected, washed 3 times with deionized water through centrifugation for 10 min at 4200 rpm speed, and finally were air-dried. The resulting polymer was pyrolyzed for 2 h at 500 °C with a 60 °C/h heating rate under an argon atmosphere. This separates copper from the nitrogenous backbone and leaves the nitrogen rings stacking around the copper species. The material is denoted as Cu/Cu_x_O-PCC-0h, indicating Cu/Cu_x_O embedded on porous carbon cuboids with no leaching (0 h). The copper content on the cuboids was then tuned via leaching. Leaching the copper species was obtained using 4 M HNO_3_. Cuboids leached for one hour were denoted Cu/Cu_x_O-PCC-1h, and the ones leached for six hours were denoted Cu/Cu_x_O-PCC-6h. Leaching was performed by immersing the carbon cuboids in the acid, followed by stirring. After leaching, the samples were thoroughly washed with deionized water before they were air-dried.

### 2.3. Material Characterization

The morphology and microstructure of the electrocatalysts were characterized with the aid of field emission scanning electron microscopy (FE-SEM, Tescan Lyra-3, TESCAN, Brno, Czech Republic) equipped with an energy-dispersive X-ray spectrometer (EDX, X-MaxN silicon drift detector, Oxford Instruments, Oxford, UK). Transmission and high-resolution transmission electron microscopes and selected area electron diffraction (TEM/HR-TEM, FEI Tecnai F20, FEI Europe B. V., Eindhoven, The Netherlands) (SAED) were used to further discern the microstructural attributes in more detail. Phase analysis was performed using X-ray diffractometry (XRD, Rigaku MiniFlex, Rigaku Co., Tokyo, Japan), and the diffractometer was operated at a 0.15416 nm wavelength, 10 mA current, and 30 kV voltage. XRD patterns were recorded from 5 to 80° in a 2θ range with 0.02° steps. The elemental state was analyzed using X-ray photoelectron spectroscopy (XPS, Thermo Scientific ESCALAB 250Xi, Waltham, MA, USA). N_2_ adsorption–desorption isotherms were collected at 77.35 K using Quantachrome Instruments (Boynton Beach, FL, USA).

### 2.4. Working Electrode Preparation

To prepare the working electrode, 5 mg of the electrocatalyst powder was added to 2 mL of ethanol. Then, 2.5 µL of Nafion solution (5 wt%) was added to act as a binder. The suspension was then sonicated for 15 min to obtain a homogeneous ink. 50 µL of the ink was deposited onto (1 cm × 1 cm) carbon paper (Fuel Cell Store). The Deposition was repeated to reach the desired catalyst loading, i.e., 0.75 mg/cm^2^. Finally, the working electrodes were allowed to dry overnight in ambient conditions.

### 2.5. Electrochemical Measurements

CO_2_ reduction experiments were conducted on a gas-tight H-type electrochemical cell. A Nafion 117 proton exchange membrane was used to separate the cathodic and anodic compartments from each other. With the aid of a potentiostat (PCI4750-38046, Gamry, Warminster, PA, USA), a three-electrode system was used to conduct the electrochemical experiments having the electrocatalyst deposited on carbon paper as the working electrode, high surface area platinum wire as the counter electrode, and an Ag/AgCl (Saturated KCl, +197 mV vs. standard hydrogen electrode) reference electrode. Working and reference electrodes were fixed in the proximity of 0.5 cm to ensure accurate measurement. All potential readings were reported against the reversible hydrogen electrode RHE (unless otherwise stated) using the following relation:

(1) $$V_{RHE\;}=V_{Ag/AgCl}+0.197+0.0592\times pH$$

As an electrolyte, 15 mL of 0.5 M KHCO_3_ aqueous solution was used in both anodic and cathodic compartments. Electrolyte pH was initially measured to be 8.4. then dropped to 7.2 as a result of purging CO_2_ for 30 min, which ensures CO_2_ saturation [42,43]. Linear sweep voltammetry LSV tests were conducted in the potential range of 0 to −1.6 V vs. Ag/AgCl at a scan rate of 50 mVs^−1^. High-purity Argon (99.999%) gas was purged into the electrolyte (cathodic side) for 30 min at 50 sccm flow rate for the Ar-saturated LSV testing. The LSV measurements for the CO_2_-saturated electrolytes were run in triplicates after purging with high-purity CO_2_ (99.999%) gas for at least 30 min at 50 sccm, and the average value was reported. Electrochemical impedance spectroscopy (EIS) was carried out under an identical experimental setup (electrodes and electrolyte), and the frequency was swapped from 10^5^–0.1 Hz at certain DC potential to record the Nyquist plots.

### 2.6. Gaseous Product Quantification

Electrolysis was carried out under control potential at ambient conditions. Prior to testing, high-purity CO_2_ (99.999%) was purged into the electrolyte (cathodic side) for at least 30 min at a 50 sccm flow rate. The flow rate was maintained at 5 sccm during reduction to ensure CO_2_ saturation conditions during the experiment. The reduction was conducted for 1 h for each potential. The cathodic compartment was connected directly to a Clarus 580 Gas Chromatograph (Perkin Elmer, Waltham, MA, USA) to analyze gaseous products. The GC was equipped with Hayesep and Molecular sieve columns; a Thermal conductivity detector (TCD) was used to analyze H_2_ gas.

A flame ionization detector (FID) with a methanizer was used to quantify hydrocarbon gases and carbon monoxide. Gas flow from the cathode headspace was sampled at 20, 40, and 60 min; the three measurements were averaged to calculate the respective faradaic efficiencies. A standard plot relating peak area to gas volume percent was used to quantify gaseous products. Obtained peak areas from the chromatogram were compared to the standard plots to find the unknown gases’ volume percent. Faradaic efficiency was calculated using the following relation

(2) $$FE\%=\frac{nzF}{It}$$

where n is the amount of detected product in moles, z is the number of electrons necessary to obtain 1 mole of product, F is the faraday constant (96,485 C/mol), t is the time required to fill the gas sampling loop (s) [44].

### 2.7. Liquid Product Quantification

Quantitative NMR spectroscopy (QNMR) was employed to quantify the yield of liquid products, such as ethanol, formate, acetate, and other compounds produced during the constant potential electrolysis. Bruker AVANCE III 9.4 T Liquid State NMR spectrometer (Bruker, Billerica, MA, USA) was used to record ^1^H NMR spectra in a single-pulse experiment. With an internal calibration standard (called as well internal reference), the absolute concentrations of the reaction products were determined. Phenol was chosen to be the internal reference since it is not structurally related to the analyte of interest, as it has resonances that do not overlap with those of the analyte and do not have excessively long T1 relaxation times [37]. ^1^H NMR was acquired by the use of a water suppression pulse sequence which is called the presaturation technique. During this pulse sequence, the water peak is suppressed by saturating its resonance frequency at δ 4.70. The acquisition parameters were time-domain data size (TD), 32,000; the number of dummy scans (DS), 8; the number of scans (NS), 128; spectral width (SW), 27.76 ppm; pulse width (pw), 90°; delay 1 (d1) and 10 s. The sample was prepared by mixing the electrolyte aqueous solution (630 µL) with deuterium oxide D_2_O (70 µL) and internal standard (30 µL of 50 mM phenol) in an NMR tube [37], [38]. The reference solution was prepared by mixing 23.5 mg of phenol (99+% purity, Aldrich) with 5 mL of high-purity deionized water. Essentially, the signals of interest integrals and the selected peak of reference (δ 7.2) and the normalization for the number of protons are needed to perform simple ratio analysis to quantify the products of interest. The molar ratio $\frac{M_{x}}{M_{y}}$ between the two compounds x and y can be determined using Formula (3), where I is the peak integral, and N is the number of nuclei giving rise to the signal.

(3) $$\;\frac{M_{x}}{M_{y}}=\frac{I_{x}}{I_{y}}\times \frac{N_{y}}{N_{x}}$$

## 3. Results and Discussion

### 3.1. Morphological and Structural Characterization

**Figure 1:**
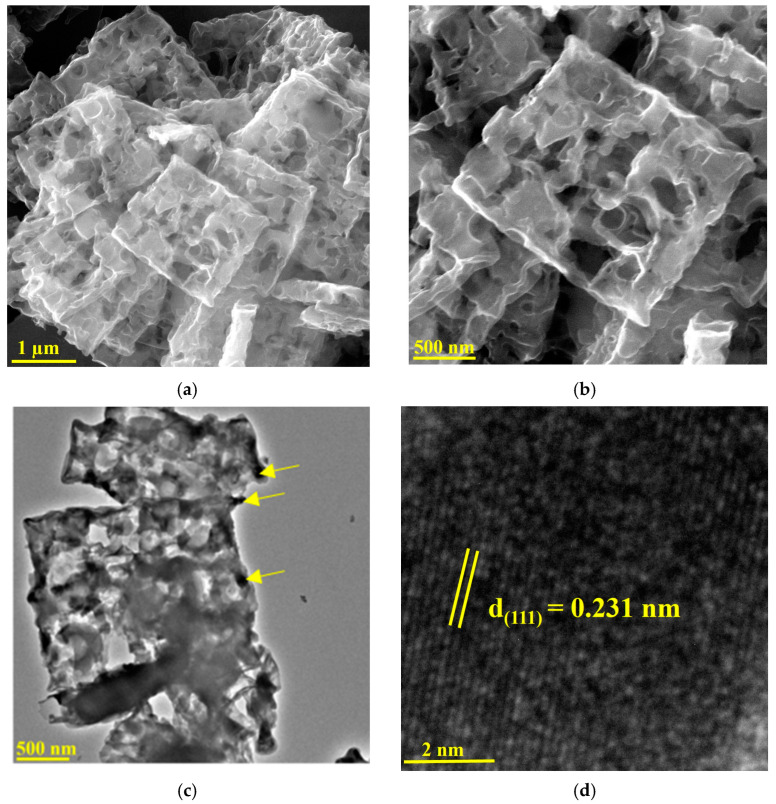

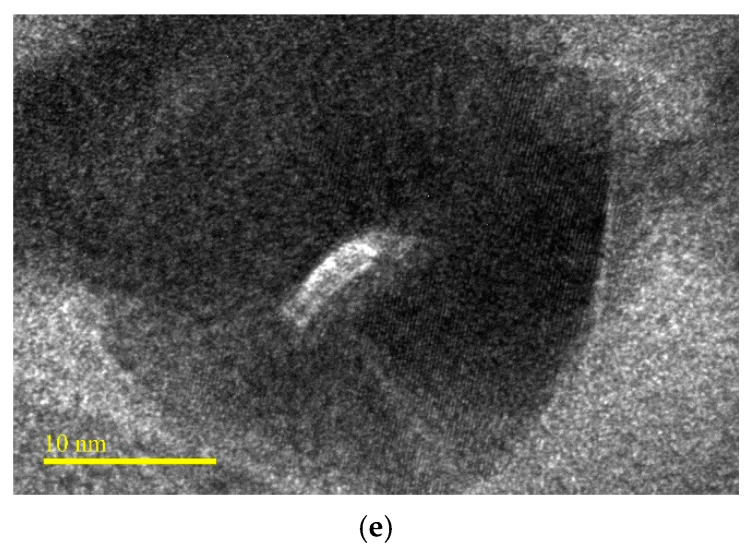
(**a**,**b**) SEM images of the unleached PCC at different magnifications, (**c**) TEM image of two overlapping cuboids, (**d**) HRTEM demonstrating lattice fringe of a copper particle, (**e**) HRTEM image of one Cu nanoparticle.

Scanning electron microscopy (SEM) was used to depict the morphological attributes of the Cu/Cu_x_O-PCC-0h at different magnifications. Figure 1a,b shows the rough, porous and amorphous nature of the overlapped and randomly aggregated cuboids. The ultrafast coordination reaction created the porous morphology, which was well maintained after pyrolysis. Measuring the dimensions of different cubes gives an average of 1.72 µm side length and 148 nm thickness (see Figure S1). The HRTEM image in Figure 1c shows two cuboids overlapping with each other. Cu/Cu_x_O nanoparticles that detached from the coordination complex after carbonization remained dispersed in the nitrogenous framework. The Cu/Cu_x_O particles appear as tiny dark dots (indicated in arrows in Figure 1c). Cu/Cu_x_O nanoparticles appear dispersed all over the cubic structure and less dispersed at some parts of the cubes. Agglomeration could be attributed to the weak physisorption of some of the nanoparticles with the carbon matrix, leading them to adhere to each other. Figure 1d shows the lattice fringes of Cu nanoparticles. The spacing of lattice fringes of one direction is found to be 0.231 nm which is close to the *d* spacing value of the (111) plane of Cu [45]. Cu/Cu_x_O nanoparticle sizes were estimated to be in a 5–30 nm size range (see Figure S2). Larger dark regions on the images represent agglomeration of the Cu/Cu_x_O particles or may appear so due to overlapped Cu/Cu_x_O-containing carbon layers. The high-resolution TEM image in Figure 1e presents one Cu nanoparticle of about 25 nm in size.

**Figure 2:**
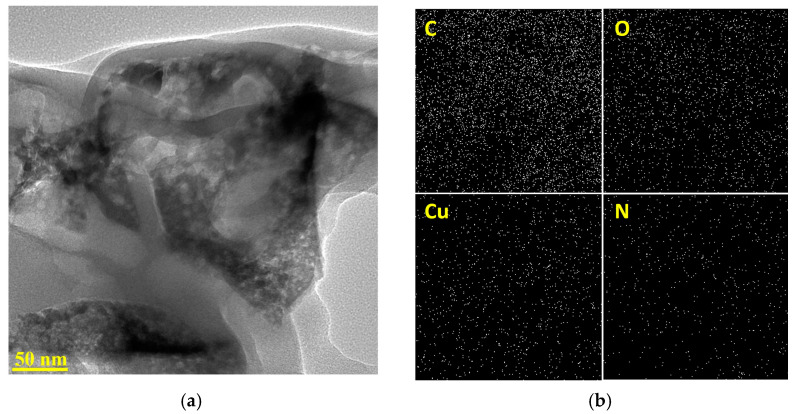
(**a**) HRTEM image of Cu/Cu_x_O-PCC-0h (**b**) elemental mapping of the Cu/Cu_x_O-PCC-0h (unleached PCC).

**Figure 3:**
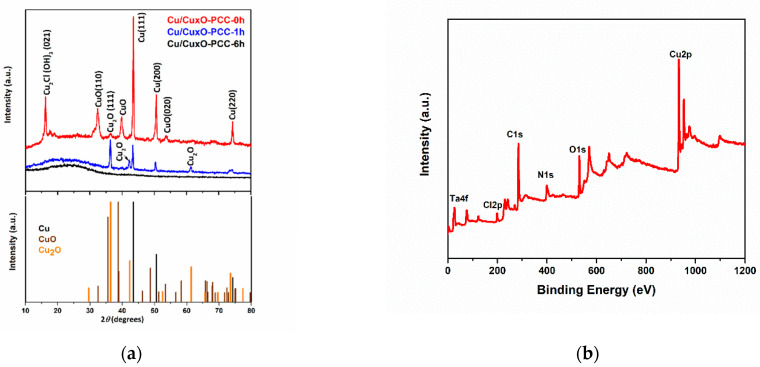

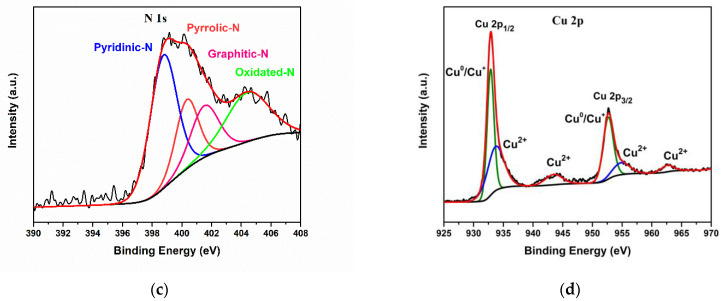
(**a**) XRD pattern, (**b**) XPS survey scan of Cu/Cu_x_O-PCC-0h, (**c**) High-resolution XPS spectrum of N 1 s for Cu/Cu_x_O-PCC-0h, (**d**) High-resolution XPS spectra of Cu 2p for Cu/Cu_x_O-PCC-0h.

Figure 2a further reveals the amorphous nature of the carbon material; rich microporosity and several multilayered graphene sheets are also apparent in the structure. Throughout the microporous structure, copper nanoparticles are well dispersed, which is also confirmed by the energy-dispersive X-ray spectroscopy elemental mapping in Figure 2b. The elemental mapping confirms the presence and uniform distribution of C, O, Cu, and N on the Cu/Cu_x_O-PCC-0h. Oxygen presence indicates the formation of copper oxides or oxygen vacancies introduced during the carbonization step. Nitrogenous species have resulted from the 4,4′-bipyridine. XPS characterization (shown in Figure 3c) is needed to see whether the pyridinic nature of nitrogen is preserved after pyrolysis. Although the overall morphology was maintained after leaching, it can be noted that the cubes on the leached materials are less confined than the unleached PCC (Cu/Cu_x_O-PCC-0h), and some of the cuboids are broken apart (see Figure S3). Cuboids in Cu/Cu_x_O-PCC-1h and Cu/Cu_x_O-PCC-6h images appear to be less dense than in Cu/Cu_x_O-PCC-0h. The EDX elemental mapping of the three materials (see Figure S3) shows a decrease in the quantity of Cu, confirming the partial leaching of copper particles. Notably, the uniform distribution of C, O, Cu, and N was still maintained after leaching. 

To further study the electrode morphology, SEM images were obtained for the bare carbon paper before and after loading with Cu/Cu_x_O-PCC-0h (see Figure S4). Bare carbon fibers appeared to be smooth and interconnected, forming connective networks. The open and vacant spaces between the fibers allow the catalyst to distribute uniformly all over the fibers. SEM images after depositing the 0.75 mg/cm^2^ show a uniform distribution of the cubes over the carbon fiber texture, i.e., cuboids overlapped with each other and stacked around the carbon fibers. After electrolysis at −0.9 V vs. RHE for 1 h, the catalyst material maintains uniform distribution on the carbon fiber texture with no change in morphology on the catalyst (see Figures S5–S8). The white sphere particles resulted from dried KHCO_3_ salt on the working electrode surface. 

XRD analysis was used to investigate the Cu/Cu_x_O crystalline structure of the three catalysts. As shown in Figure 3a, peaks located at 2θ = 43.4, 50.5, and 74.2 degrees are distinct peaks of Cu^0^ at planes (111), (200), and (220), respectively [46]. Cu/Cu_x_O-PCC-0h has those peaks sharper than Cu/Cu_x_O-PCC-1h. Clearly, no peaks were recorded for Cu/Cu_x_O-PCC-6h due to very tiny amounts of Cu. The XRD results confirmed the presence of CuO (32.5°, 39.9°, 53.5°) [47] and Cu_2_O (36.5°, 42.4°, 61.9°) [47] in Cu/Cu_x_O-PCC-0h and Cu/Cu_x_O-PCC-1h catalysts. The oxides were probably formed during carbonization and during air drying.

The higher quantity of Cu_2_O on Cu/Cu_x_O-PCC-1h could be attributed to air oxidation after leaching or to a less preferably leaching effect for Cu_2_O; lower Cu^0^ intensity in Cu/Cu_x_O-PCC-1h is not only due to leaching of copper but also due to oxidizing of residual Cu by the nitric acid. Higher intensity peaks of Cu^0^ indicate that Cu^0^ is most abundant in Cu/Cu_x_O-PCC-0h but not in the partially leached catalyst. Cu_2_O (111) was observed to be a preferred orientation for the Cu_2_O crystallites in the Cu/Cu_x_O-PCC-1h, thus having higher intensity. It is worth noting that in the Cu/Cu_x_O-PCC-1h catalyst, Cu_2_O is more soundly present with a small amount of elemental Cu. This does not necessarily mean that Cu_2_O formed preferably due to acid/air oxidation; rather, it indicates that Cu_2_O is more difficult to leach as it is more strongly adsorbed to the carbon matrix.

The absence of CuO in the partially leached copper indicates the leaching of most CuO particles. In the unleached carbon cubes, the peak at 16.22° angle is attributed to the diffraction of another Cu species; it can be indexed to copper chloride hydroxide Cu_2_Cl (OH)_3_ [48]. The broad peak in the region from 10 to 30° is distinct for the amorphous structure of the carbon, as evidenced in Ref. [41]. The crystallite size of Cu^0^ in Cu/Cu_x_O-PCC-0h was calculated by the Scherrer equation and found to be around 23.37 nm (it was calculated by averaging the particle size obtained from the three distinct Cu peaks).

(4) $$D=\frac{K\lambda }{W_{FWHM}cos\theta }\;\;\;\;\;$$

where D is the crystallite size, K is a constant, λ is the wavelength, and $W_{FWHM}$ is the full width at half maximum of the taken peak, and θ is the diffraction angle in radians.

This result coincides well with the particle sizes obtained from HRTEM images. d spacing for the Cu (111) plane was calculated to be 0.208 nm which is in good agreement with the value obtained from HRTEM images.

**Figure 4:**
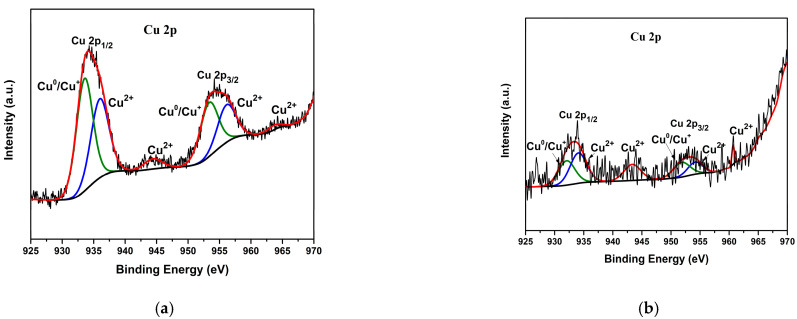
High-resolution XPS spectra of Cu 2p for (**a**) Cu/Cu_x_O-PCC-1h and (**b**) Cu/Cu_x_O-PCC-6h.

The surface chemistry and elemental composition of the Cu/Cu_x_O-PCC-0h catalyst were analyzed using XPS. The binding energies of all present elements were normalized to that of C_1s_ at 284.8 eV. The survey spectrum in Figure 3b confirms the EDX analysis showing the characteristic peaks of C_1s_, N_1s_, O_1s,_ and Cu_2p_ located at 248.8, 402.2, 531.3, and 931.2 eV binding energies, respectively. There are negligible peaks of Ta_4f_, which were associated with the foil used for depositing the sample, and a small Cl_2p_ peak from Cu_2_Cl (OH)_3_ species. Investigation of the oxidation states of Cu through fitting main and satellite peaks (see Figure 3d) shows the presence of the two copper oxidation states, Cu^+^ and Cu^2+^, along with Cu^0^. The major peaks present at 932.86 and 952.66 eV correspond to Cu 2p_3/2_ and Cu_2p1/2_, respectively. Those two major peaks’ positions indicate their origination from Cu^0^/Cu^+^, representing the majority of the present copper; this agrees well with the XRD analysis in Figure 3a. Adjacent peaks at 933.69 and 954.85 eV correspond to Cu^2+^ (cupric oxide) [49]. Cu^2+^ existence is also indicated by the presence of satellite peaks at higher binding energies from the main peaks, i.e., at 943.51 and 962.6 eV [50]. The deconvolution of N_1s_ spectra, presented in Figure 3c, reveals the presence of four nitrogen groups commonly observed in nitrogen-containing materials. Those nitrogen groups are pyridinic, pyrrolic, graphitic, and oxidated nitrogen [51]. Here, they are observed at the binding energies of 398.76, 400.34, 401.52, and 404.41 eV, respectively. Peak positions of each type of nitrogen configuration may slightly differ from other studies due to changes in the environments of nitrogen, the charge of nitrogen, and its bonded atoms [52]. Figure 4a,b shows the Cu_2p_ core level region for Cu/Cu_x_O-PCC-1h and Cu/Cu_x_O-PCC-6h. There is a clear depletion of copper content upon leaching, with Cu/Cu_x_O-PCC-6h having the least amounts. Despite XRD spectra, peaks of copper oxides were apparent in the X-ray spectroscopy results due to the higher sensitivity of XPS. High-resolution XPS spectra for C_1s_, N_1s_, and survey scans for the three catalysts are presented in Figure S5.

The textural characteristics of the three catalysts were also investigated. Table 1 presents the BET surface area, micropore volume (< 2 nm), mesopore volume (2–50 nm), and total pore volume of the three samples. Obviously, the BET surface area was positively correlated with the catalyst leaching time, with around 175% and 262% increase in the BET surface area upon one hour and six hours of leaching, respectively. The observed increase in the surface area upon leaching can be ascribed to the introduction of more pores into the nitrogenous carbon backbone, which was evidenced by the higher total pore volume. 

Notably, the leaching step leads to more micropores than mesopores. This is due to disconnecting Cu/Cu_x_O nanoparticles from the carbon-nitrogen network leaving microporous defects in the structure.

**Figure 5:**
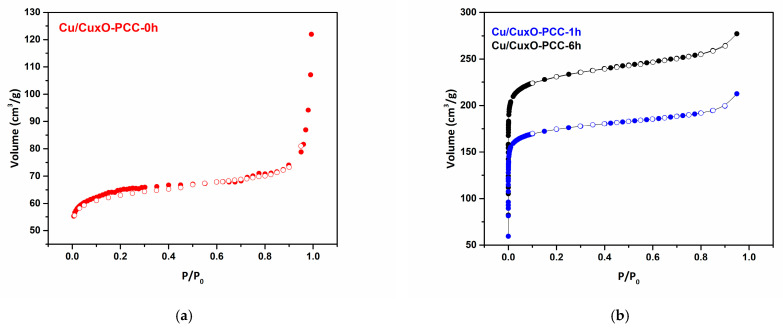
(**a**) adsorption-desorption isotherm of the unleached PCC catalyst, Cu/Cu_x_O-PCC-0h (**b**) adsorption-desorption isotherms of the leached PCC catalysts, Cu/Cu_x_O-PCC-1h, Cu/Cu_x_O-PCC-6h.

**Figure 6:**
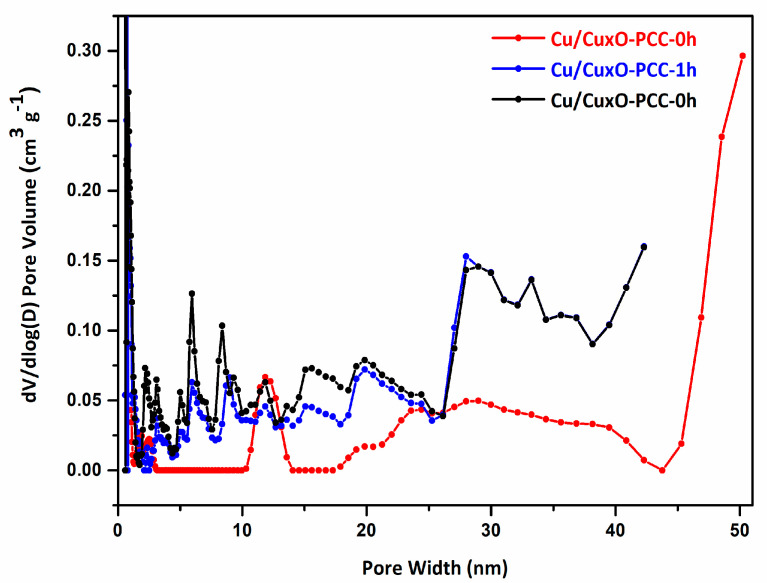
DFT pore size distribution for the three catalysts.

Nitrogen adsorption–desorption isotherms were performed to determine the textural properties of the three samples displayed in Figure 5a,b. Partially leached samples Cu/Cu_x_O-PCC-1h and Cu/Cu_x_O-PCC-6h showed type I isotherms without any hysteresis loop. At low relative pressure (P/P_0_ < 0.01), the isotherms show steep N_2_ uptake indicating rich microporosity. At the mid-range relative pressure, slight volume uptake can be observed due to the existence of mesopores. The existence of mesopores is also shown in the pore size distribution calculated by the DFT model, Figure 6. The slight volume change in the high relative pressure region resulted from the existence of mesopores in the structures. Comparing the isotherms of the partially leached catalysts shows that Cu/Cu_x_O-PCC-6h has higher N_2_ volume uptake than Cu/Cu_x_O-PCC-1h, which displays the effectiveness of leaching time on enriching the microporosity. Cu/Cu_x_O-PCC-0h, on the other hand, displays a slightly different character from that of the leached samples. The moderate adsorption in the low relative pressure region, the slight hysteresis loop, and the abrupt change at the high relative pressure region all indicate that mesopores are more abundant in the structure compared to the leached samples. However, micropores deriving from carbonization are still the dominant pore type.

The DFT pore size distribution illustrated in Figure 6, shows the different distribution between the unleached and partially leached samples. The partially leached samples (Cu/Cu_x_O-PCC-1h and Cu/Cu_x_O-PCC-6h) depict similar pore size distributions indicating pores with the same nature. The leached samples display greater variety in pore sizes compared to the unleached sample. The variation in the pore sizes results from variation in the Cu/Cu_x_O nanoparticles. Among the three catalysts, Cu/Cu_x_O-PCC-0h has the highest proportion of pores in the size range of 10–14 nm. The relationship between pore size and particle distribution is rather indirect. The uniform distribution of pores over the mass of the carbon suggests more distribution of the nanoparticles. Cavities have more functionalities and active space to host metal nanoparticles. This is of great importance to minimize metal aggregation and to exploit the catalytic properties of the hybrid material. Pore size also has an indirect connection with particle distribution. When the pores are stable and controlled in size (same size), the material can be reproduced with the same properties. In the catalytic reaction of the Cu/Cu_x_O nanoparticles in the porous carbon templates, stable size and distribution of the nanoparticles are expected to improve their performance.

### 3.2. Electrocatalytic Reduction of CO_2_

**Figure 7:**
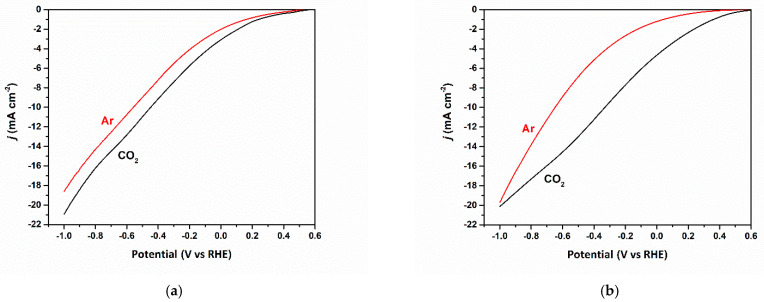

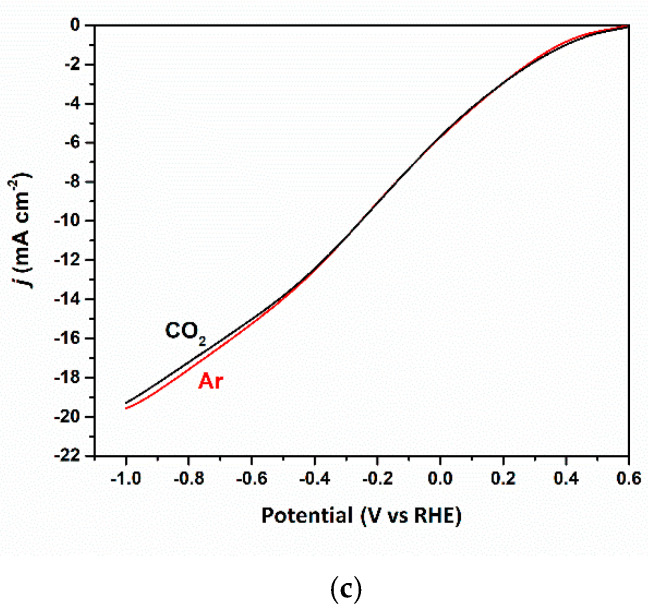
(**a**–**c**) LSV scans under Ar and CO_2_ saturated electrolytes for Cu/Cu_x_O-PCC-0h, Cu/Cu_x_O-PCC-1h, and Cu/Cu_x_O-PCC-6h, respectively.

To assess the electrochemical performance of the three catalysts, Linear sweep voltammetry (LSV) measurements were conducted. Figure 7a–c compares the LSV plots of the three catalysts under Argon and CO_2_ saturated electrolytes. The three catalysts display a monotonic increase in current densities upon increasing the overpotentials. The increased cathodic current density in Ar-saturated electrolytes is most probably attributed to the hydrogen evolution reaction HER. However, for the CO_2_-saturated media, the increased cathodic current density resulted from both HER and CO_2_ reduction. In Figure 7a, the higher current densities in CO_2_-saturated electrolytes indicate more reactivity to CO_2_ reduction over HER. Starting at 0.4 V vs. RHE, CO_2_ reduction starts to display higher reactivity over HER.

The Cu/Cu_x_O-PCC-0h catalyst displays an extremely low onset potential of about 0.4 V vs. RHE; the increment rate of current density increases after the 0.2 V vs. RHE, then it increases more at −0.2 V, after which the same rate is maintained. Although very small, it is worth mentioning that the reduction peak at 0.45 V vs. RHE can be ascribed to reduced CuO to Cu_2_O [53]. While the LSV behavior for the Cu/Cu_x_O-PCC-1h catalyst is similar to that of Cu/Cu_x_O-PCC-0h under CO_2_ saturated electrolyte, argon saturated electrolyte displays different behavior. The gap between both Ar- and CO_2_-saturated LSVs (see Figure 7b) increases gradually and reaches a maximum at −0.5 V before decreasing until meeting at one point at −1.0 V vs. RHE. The peak at 0.45 V vs. RHE disappears since CuO almost disappeared after leaching, as observed from XRD. Cu/Cu_x_O-PCC-6h catalyst in Figure 7c, however, shows similar behavior in both environments, which can be attributed to the lower quantity of CO_2_ favoring active sites.

**Figure 8:**
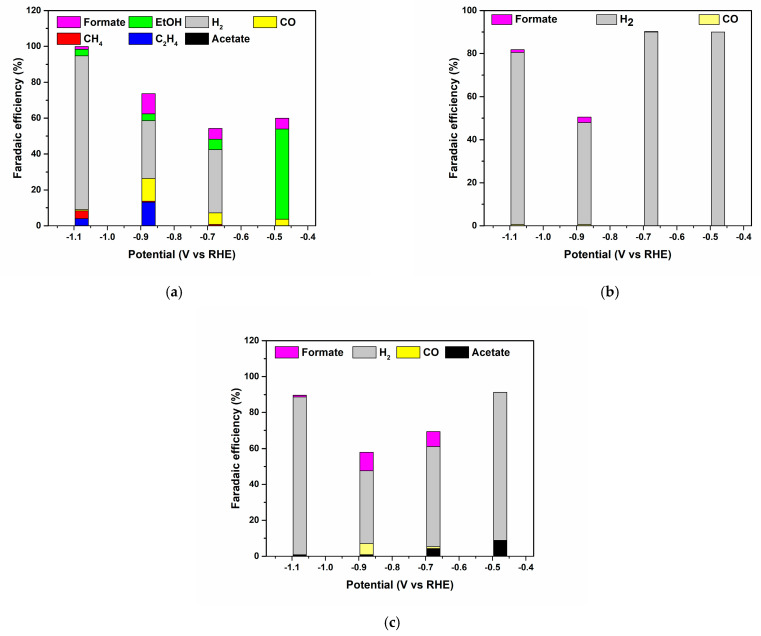
(**a**–**c**) Faradaic efficiencies of different products against the potential for Cu/Cu_x_O-PCC-0h, Cu/Cu_x_O-PCC-1h, and Cu/Cu_x_O-PCC-6h, respectively.

Controlled potential electrolysis experiments were conducted in an H-type cell in a CO_2_-saturated 0.5 M KHCO_3_ solution to evaluate Faradaic Efficiencies (Fes) of both gaseous and soluble products. Fes were evaluated at six different potentials starting from −0.1 V up to −1.1 V vs. RHE. At the lowest potential range of −0.1 and −0.3 V, no products were observed for the three catalysts. However, at the potential of −0.5 V vs. RHE, products started to appear in the three catalysts. The onset potential for CO_2_ reaction is most likely to be in the range of −0.4–0.5 V vs. RHE. Although LSV results suggest lower onset, detectable quantities were only observed at this potential range. For Cu/Cu_x_O-PCC-0h (see Figure 8a), the detected liquid products from H^1^NMR were acetate, formate, and ethanol, whereas the detected gaseous products were hydrogen, carbon monoxide, methane, and ethylene. This variety in products is commonly observed in Cu-containing catalysts [54].

At the lowest tested potential −0.5 V vs. RHE, the detected ethanol quantity resulted in 50% FE. This corresponds to 77% selectivity for ethanol from CO_2_ reduction products. Ethanol production at low overpotentials was reported to be generated on Cu/Cu^+^-containing composites [35,36]. Produced hydrogen amounts were undetectable by the GC, indicating clear HER suppression. As potential was increased to −0.7 V, ethanol FE decreased to 5.8%. This decrease in ethanol FE is accompanied by an increase in HER to 35% and an increase in CO FE to 6.3%. Trace amounts of methane and acetate were also observed at −0.7 V vs. RHE.

In a potential of −0.9 V, at which several studies reported selectivity to ethylene [10,13,55], an FE of 13% was observed. Formate and CO had their peak Fes at this potential in their volcano trend behavior. At the highest tested potential −1.1 V vs. RHE, there is a significant increase in hydrogen Fes accompanied by an increase in the current density (see Table S1). Regarding hydrocarbons at this potential, a drop of ethylene FE to 4% along with the appearance of 4% methane FE was observed.

Leaching the catalyst for 1 h (Figure 8b) allows the catalyst to favor HER over the CO_2_ reduction reaction. Although LSV results indicated the favorability of CO_2_ reduction, this observation indicates that the increase in cathodic current density in the CO_2_-saturated electrolyte has a good contribution from HER. The increased current density in the CO_2_-saturated solution is, therefore, mainly due to the HER. Because the pH values of CO_2_- and Ar-saturated environments differ, the nature of the HER may be preferred in the CO_2_-saturated environment’s higher acidic pH [36]. However, acetate can be observed at low quantities of 8.8% at a potential of −0.5 V vs. RHE. Increasing the potential to −0.7 V reduces the acetate FE to 4%, which is similar to the observed trend for ethanol in Cu/Cu_x_O-PCC-0h.

**Figure 9:**
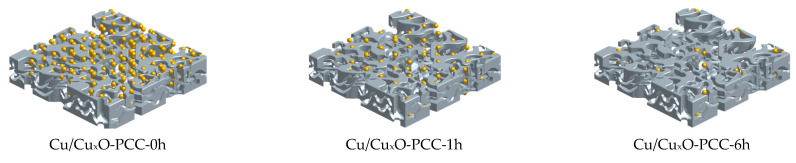
Schematic representation of the three catalysts.

This behavior suggests that the change in Cu/Cu_x_O-PCC-1h selectivity is mostly due to a considerable decrease in the amount of Cu/Cu_x_O nanoparticles having to note that leaching does not affect the size of the nanoparticles. Further leaching of available Cu/Cu_x_O particles in the cubic structure hindered the activity of the catalyst towards CO_2_ reduction (as can be seen in Figure 8c) and altered its reactivity to water splitting instead. This further supports the fact that Cu/Cu_x_O nanoparticles served as the active sites on those materials. Furthermore, it indicates that nitrogen species did not directly serve as the main active site to CO_2_ reaction but rather facilitated CO_2_ reduction. The mere CO_2_ reaction selectivity at the highest potential in the three catalysts indicates the transformation of the nature of the electrochemical reaction to being a diffusion-limited reaction at higher overpotentials. Electrons became more abundant inside the structure and conducted efficiently throughout the material, but they reacted faster with the positively charged protons rather than CO_2_. However, at low potentials, adequate CO_2_ diffusion is obtained, allowing for the stability of reaction intermediates, resulting in C-C coupling, as shown in earlier investigations [36]. The scheme in Figure 9 visualizes the difference in Cu amount in the three catalysts.

**Figure 10:**
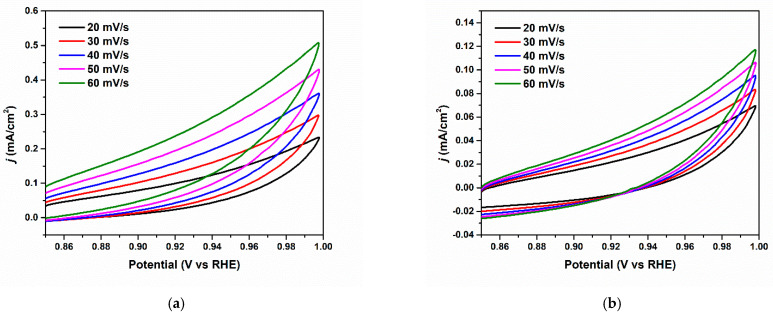

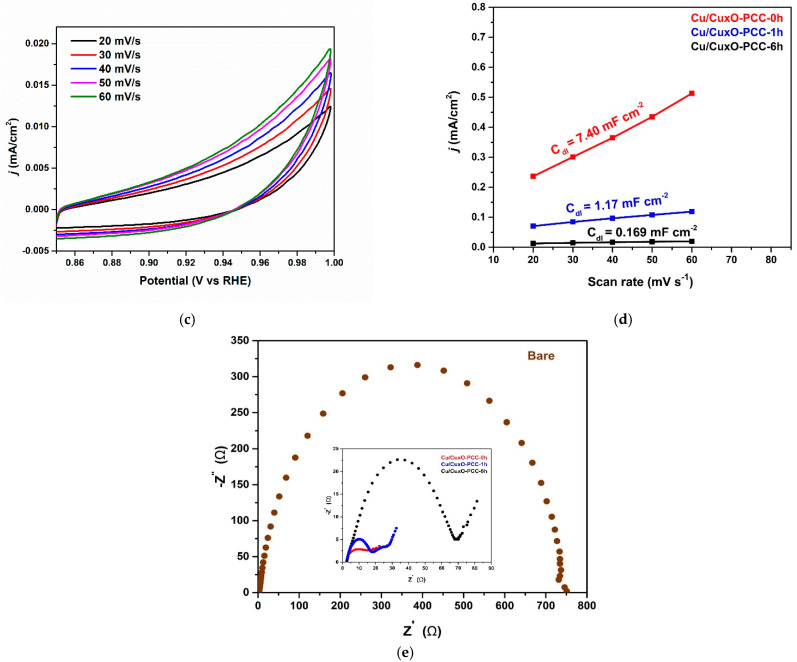
(**a**–**c**) CV plots electrochemical double-layer measurement electrolytes for Cu/Cu_x_O-PCC-0h, Cu/Cu_x_O-PCC-1h, and Cu/Cu_x_O-PCC-6h, respectively. (**d**) Electrochemical double layer plot (**e**) Nyquist plots at −0.5 V vs. RHE.

The higher intrinsic activity of the Cu/Cu_x_O-PCC-0h might be partially ascribed to the higher electrochemical active surface area (ECSA). Cyclic voltammetric scans were applied at different scan rates in a CO_2_-saturated 0.5 M KHCO_3_ solution (Figure 10a–c). In Figure 10d, the respective capacitive currents were plotted against scan rates to evaluate the double-layer capacitance C_dl_, which is positively correlated to the ECSA. Cu/Cu_x_O-PCC-0h displayed the highest value of 7.4 mF cm^−2^ compared to the partially leached catalysts, which showed 1.17 and 0.169 mF cm^−2^ for Cu/Cu_x_O-PCC-1h and Cu/Cu_x_O-PCC-6h, respectively. This corresponds to an 85% and 98% decrease in ECSA upon leaching for 1 and 6 h, respectively. The presence of well-distributed active sites, in addition to the synergetic effect of Cu/Cu_x_O nanoparticles with nitrogen-carbon hybrid porous support, resulted in an increase in ECSA. This demonstrates the effectiveness of anchoring Cu/Cu_x_O nanoparticles in porous carbon cuboids, as well as the dependence of CO_2_ reactivity on the number of Cu nanoparticles.

To further investigate the electronic properties of the catalyst, electrochemical impedance spectroscopy (EIS) test was performed under CO_2_-saturated 0.5 M KHCO_3_ solution at a potential of −0.5 V vs. RHE from 10^5^ Hz to 0.1 Hz. The resultant Nyquist plots are shown in Figure 10e. As depicted in Figure 10b, the bare electrode has a larger semicircle diameter representing larger charge transfer resistance (R_ct_). Depositing the catalyst material into the bare electrode changes the electronic characteristics of the electrode. Bare electrode resistance is mainly due to the charge transfer at the electrode/electrolyte interface while adding the catalyst significantly reduces this resistance and shows more contribution to the ohmic resistance of the electrolyte. The inset of Figure 10e shows Nyquist plots for the three samples; charge transfer resistance (R_ct_) is shown to be strongly correlated to the number of Cu/Cu_x_O nanoparticles. Notably, the reported electrocatalysts in this study are considered to be among the best-reported electrocatalyst materials for the electrochemical conversion of CO_2_ (see Table 2).

Although the BET surface area of the Cu/Cu_x_O-PCC-0h is relatively lower than the partially leached catalysts, it displays higher double-layer capacitance and, therefore, more electrochemically active surface area. The higher BET surface area of the partially leached samples arises from leaching the Cu/Cu_x_O nanoparticles and the creation of more vacancies in the heterogeneous system, but this area is not electrochemically active or accessible towards electrochemical reactions as for their higher charge transfer resistance. This was supported by the decrease in the current density at all potentials upon leaching. For instance, the current density at −0.5 V vs. RHE decreased by 36% and 47% upon leaching for 1 and 6 h, respectively (see Table S1). Indeed, more control experiments are needed to elucidate the effect of porosity as, here, porosity is created at the expense of CO_2_ reduction active sites. However, it is worth mentioning that the distribution of pores on the Cu/Cu_x_O-PCC-0h tends to be more mesoporous in nature than the partially leached catalysts, which are mainly microporous and affect the functionality of nitrogen species. N-doped mesoporous carbon materials in previous reports proved to promote CO_2_ conversion to ethanol [61]. Moreover, larger pore sizes and pore volumes are shown to enhance nitrogen functionalization by which efficient adsorption of CO_2_ can be achieved [62]. As such, higher electron transfer resistance observed in the leached samples could be attributed to the less effective functionalization of nitrogen. The significant effect of Cu/Cu_x_O amounts present in the material proves the effectiveness of the porous carbon support material as they offer easy exposure of CO_2_ and electrolyte molecules to the active sites. These results partially assert the fact that controlling the micro and meso interfaces and morphology of porous carbon support materials plays an important role in material behavior in many applications including electrochemical CO_2_ conversion [63].

**Figure 11:**
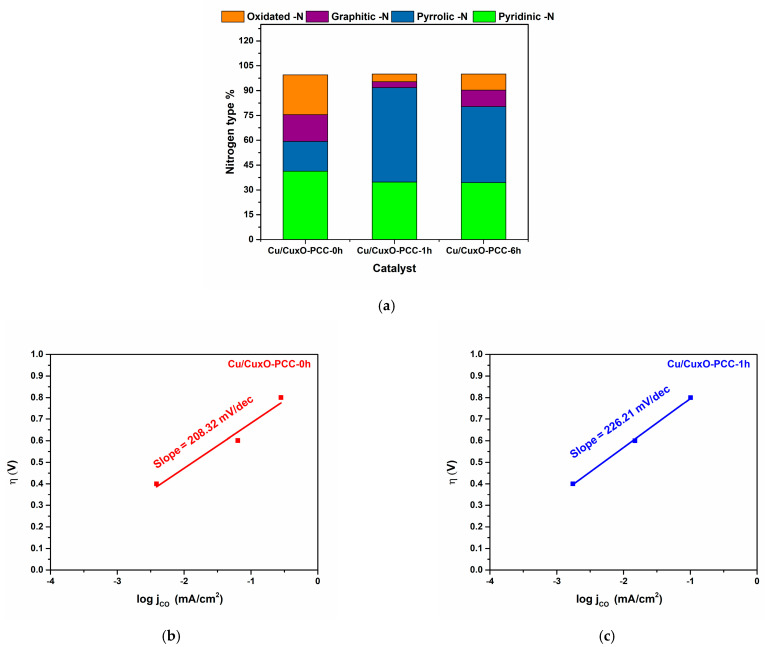
(**a**) Relative amounts of nitrogen species in the three samples, (**b**,**c**) Tafel plots of CO on Cu/Cu_x_O-PCC-0h and Cu/Cu_x_O-PCC-1h catalysts.

Previous reports have shown a high dependence of catalyst activity on the Cu amount in the catalyst [57]. The presence of higher quantities of pyridinic and graphitic nitrogen species, shown in Figure 11a in the Cu/Cu_x_O-PCC-0h, also worked as a facilitator to CO_2_ reduction with C-C coupling reaction mainly occurring on the Cu/Cu_x_O active sites. Other reports strongly support the fact that selectivity to C_2_ products is simultaneously ascribed to the introduction of copper and nitrogen systems on carbon-supported materials.

Looking at the reaction mechanism, adsorbed CO_2_ is firstly chemisorbed and stabilized. Hydrogenation of the chemisorbed CO_2_ through *H or H^+^ protons generates *COOH intermediate. The formation of *CO proceeds through the dehydrogenation of *COOH. Generally, *CO is an important intermediate towards C_2_ product generation. *CO can either desorb from the catalyst or further react to form more complex products. Examining some inherent properties of the catalysts towards CO formation mechanism and kinetics would help explain their performances. Tafel plots towards CO formation on the Cu/Cu_x_O-PCC-0h and Cu/Cu_x_O-PCC-1h catalysts showed linear fit in the tested potential region, excluding the highest potential value. Slope values of the linear fits displayed 208.32 mV/dec and 226.21 mV/dec for Cu/Cu_x_O-PCC-0h and Cu/Cu_x_O-PCC-1h, respectively (see Figure 11b,c).

Slopes closer to 118 mV/dec indicate the adsorption of CO_2_ along with the single electron transfer step to CO_2_ as the rate-determining step, while slopes closer to 59 mV/dec indicate another rate-determining step occurring after the single electron transfer step [57,64]. Higher slopes than 118 mV/dec indicate slower kinetics towards CO intermediate formation. However, relatively speaking, the Cu/Cu_x_O-PCC-0h catalyst shows ≈9% slope decrease, indicating faster kinetics in forming *CO, which is an important intermediate towards the formation of C_2_ products [11]. The faster kinetics of Cu/Cu_x_O-PCC-0h can be attributed to its surface energy which provides a more optimum reaction condition for the *CO intermediate. According to the Sabatier principle, the *CO intermediate shall not bind too weakly to be able to react further nor too strongly not to poison the catalyst surface [65]. Edge-sited pyridinic-N molecules might offer a lower energy barrier towards *CO_2_ with an optimal binding strength for *COOH generation to be able to further dimerize and continue the ethanol formation path journey [66].

These results support the findings in [67], which concluded that the pyridinic type of nitrogen not only plays an important role in decreasing the overpotentials of CO_2_ reduction reaction but also increases selectivity towards CO, which is an important intermediate towards ethanol formation. Pyridinic defects also were demonstrated to have lone pairs of electrons capable of binding CO_2_, and they have also proven to be effective agents for CO_2_ reduction in different studies [67].

Dimerization or C-C coupling has been reported to occur in Cu hybrid systems. Hybrid Cu systems with carbon–nitrogen structures attain strong electronic properties and provide more electrons and adsorbed hydrogen species that accelerate the hydrogenation of *CO. This is expected to create high local pressures of CO_2_ around the porous carbon structure, which enhances the formation of the C-C couples [68]. Looking at the diminished selectivity towards ethanol on the partially leached catalysts strongly supports the hypothesis that there is an optimum valence Cu state, particle size, and metal amount that allow favorable interaction with the functionalized carbon structure towards higher certain C_2_ product selectivity. Moreover, the nature of the pores, pore distribution, and pore size are key design parameters for high electrochemical activity. The co-existence of micro- and mesopores seems to be favorable for the mass transport of CO_2_ into the anchored Cu/Cu_x_O nanoparticles. The presence of different copper states along with metallic copper seems to be beneficial for CO_2_ reaction; Cu^+^ presence provides strong adsorption of O atoms onto the cation centers, which lowers ethanol production overpotential.

DFT calculations performed by Y. Song et al. [58] revealed sufficient catalytic sites on Cu nanoparticles placed on highly textured nitrogen-doped carbon nanospikes. These sites are excellent adsorbents for *OCCO intermediate, which promotes ethanol generation. Moreover, for the C-C coupling acceleration on Cu nanoparticles embedded in the N-doped carbon layer, the catalyst simultaneously inhibited the breakage of the C-O bond in the *HOCCH intermediate [69]. Overall, more evidence in this work indicates that Cu/Cu_x_O is the main active component site for forming the C-C bond considering the diminished CO_2_ reactivity on the leached catalysts.

Leaching the Cu/Cu_x_O-PCC-0h catalyst not only reduced the amount of Cu/Cu_x_O, which is considered to be the main driver for CO_2_ conversion into ethanol, but also changed the surface chemistry and morphology of the catalyst. As illustrated in Figure 7a, the amount of pyrrolic-N was increased at the expense of graphitic-N, and relatively fewer pyridinic-N species were present in the structure. Leaching with acids is known to introduce several oxygen functional groups into the carbonaceous system [70]; as such, Cu/Cu_x_O-PCC-6h contains more oxygen functional groups than the rest of the catalysts. A more in-depth study on the catalytic role of oxygen functional groups is needed. However, oxygen functional groups proved to have a synergetic role in CO_2_ electrochemical reduction. The favorability of HER at low overpotentials was noted on the Ag catalyst supported on CNT enriched with -COOH functional groups. The study also proved the difference in ECSA upon inducing different oxygen functional groups [71]. Suppression of HER in Cu/Cu_x_O-PCC-0h and its emergence on Cu/Cu_x_O-PCC-1h and Cu/Cu_x_O-PCC-6h catalysts at the low potential range may be partially attributed to the presence of different oxygen functional groups on the carbon support.

## 4. Conclusions

A series of novel porous carbon cuboids with a wide range of BET surface area (250.9–909.2 m^2^ g^−1^), total pore volume (0.189–0.430 cm^3^ g^−1^), and different amounts of Cu/Cu_x_O nanoparticles were synthesized and evaluated against electrochemical CO_2_ reduction. Notably, the developed carbon-based catalysts demonstrated considerable electrocatalytic dependence on the quantity of Cu/Cu_x_O nanoparticles. At low overpotential (~0.59 V), a maximum of 50% FE towards ethanol was achieved in the (17 Cu at%) porous cuboid carbon. The presence of more pyridinic nitrogen (41.3 at%) along with multiple Cu/Cu_x_O oxides is thought to assist efficient CO_2_ conversion. Accessible channels provided by the porous morphology facilitated the mass transport of the reactants to the anchored Cu/Cu_x_O active sites. This work proves the viability of porous carbon composite structures as an efficient support material. This work can pave the way towards further elucidating the mechanism of CO_2_ conversion on carbon-based electrocatalyst materials and determining the synergistic effects of carbon-based/Cu composite structure.

## Figures and Tables

**Table 1:** BET surface area and pore volume of the three catalysts.

Catalyst	BET Surface Area [m^2^ g^−1^]	Total Pore Volume [cm^3^ g^−1^]	Mesopores Pore Volume [cm^3^ g^−1^]	Micropores Pore Volume [cm^3^ g^−1^]	Pore Width [nm]
Cu/Cu_x_O-PCC-0h	250.9	0.189	0.089	0.100	50.232
Cu/Cu_x_O-PCC-1h	689.5	0.330	0.091	0.239	0.545
Cu/Cu_x_O-PCC-6h	909.2	0.430	0.117	0.313	0.545

**Table 2:** Comparison of CO_2_ reduction to ethanol with different types of electrocatalysts from the literature.

Catalyst	Electrolyte	Faradic Efficiency%	Potential [V vs. RHE]	Ref.
Cu/Cu_x_O nanoparticles embedded on porous carbon cuboids	0.5 M KHCO_3_	50	−0.5	This work
Oxide-derived Cu/C catalysts by facile carbonization of Cu-based MOF	0.1 M KHCO_3_	35	−0.5	[36]
Cu/Cu_2_O nanocomposite loaded on the surface of carbon ZIF-L coated on GO	0.5 M KHCO_3_	70.52	−0.87	[35]
Cobalt oxide anchored on N-doped Mesoporous carbon and CNTs	0.5 M KHCO_3_	60.1	−0.32	[56]
Cu nanoparticles decorated on pyridoxine modification graphene oxide sheets	0.1 M KHCO_3_	56.3	−0.25	[57]
Copper nanoparticle ensembles	0.1 M KHCO_3_	16.6	−0.86	[14]
Nanoflowers and nanosheets with Cu foam as a substrate	1 M KHCO_3_	35.7	−0.4	[16]
Cu nanoparticles on highly textured nitrogen-doped carbon nanospike	0.1 M KHCO_3_	63	−1.2	[58]
N-doped nanodiamonds and Cu nanoparticles	0.5 M KHCO_3_	28.9	−0.6	[59]
Carbon-supported Cu catalyst synthesized by an amalgamated Cu–Li method	0.1 M KHCO_3_	91	−0.7	[60]

## Data Availability

Not applicable.

## Supplementary Materials

The following supporting information can be downloaded at: https://www.mdpi.com/article/10.3390/nano13020230/s1, Figure S1: SEM image of the unleached PCC with side dimensions; Figure S2: HRTEM images of one copper particle; Figure S3: SEM images and elemental mapping of the three catalysts together; Figure S4: SEM images for the carbon paper before loading, after loading the catalyst and after the reaction; Figure S5: XPS spectra for the three catalysts; Table S1: Current density values for the three catalysts at different potentials; Figure S6: NMR spectrum showing Ethanol produced at −0.5 V vs. RHE; Figure S7: Electrochemical Cell and working electrode during electrolysis; Figure S8: Working Electrodes; Table S1: current densities at different potentials.
