# Supplementary material for: Low Overpotential Electrochemical Reduction of CO2 to Ethanol Enabled by Cu/CuxO Nanoparticles Embedded in Nitrogen-Doped Carbon Cuboids
Source: Nanomaterials (Basel). 2023 Jan 4;13(2):230. doi: 10.3390/nano13020230 (PMC9863918; doi:10.3390/nano13020230)
Supplement: Supplementary file 1 [file nanomaterials-13-00230-s001.zip › nanomaterials-2130214-supplementary.pdf]

Supporting Information

Low Overpotential Electrochemical Reduction of CO_2 to Ethanol Enabled by Cu/Cu_xO Nanoparticles Embedded in Nitrogen-Doped Carbon Cuboids

Monther Q. Alkoshab [1], Eleni Thomou [2], Ismail Abdulazeez [3], Munzir H. Suliman [4], Konstantinos Spyrou [2], Wissam Iali [5,6], Khalid Alhooshani [5] and Turki N. Baroud [3,7,*]

1 Department of Mechanical Engineering, King Fahd University of Petroleum & Minerals, Dhahran 31261, Saudi Arabia
2 Department of Materials Science and Engineering, University of Ioannina, 45110 Ioannina, Greece
3 Interdisciplinary Research Center for Membranes and Water Security, King Fahd University of Petroleum & Minerals, Dhahran 31261, Saudi Arabia
4 Interdisciplinary Research Center for Hydrogen and Energy Storage (IRC-HES), King Fahd University of Petroleum & Minerals (KFUPM), Dhahran 31261, Saudi Arabia
5 Department of Chemistry, King Fahd University of Petroleum & Minerals, Dhahran 31261, Saudi Arabia
6 Interdisciplinary Research Center for Refining and Advanced Chemicals, King Fahd University of Petroleum and & Minerals, Dhahran 31261, Saudi Arabia
7 Department of Materials Science and Engineering, King Fahd University of Petroleum & Minerals, Dhahran 31261, Saudi Arabia
* Correspondence: turkibaroud@kfupm.edu.sa

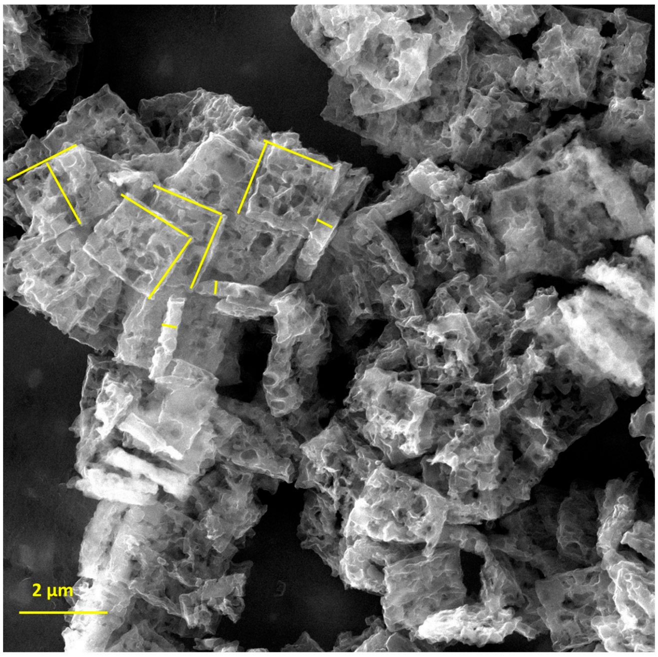

Figure S1: SEM image of the unleached PCC with side dimensions.

a)

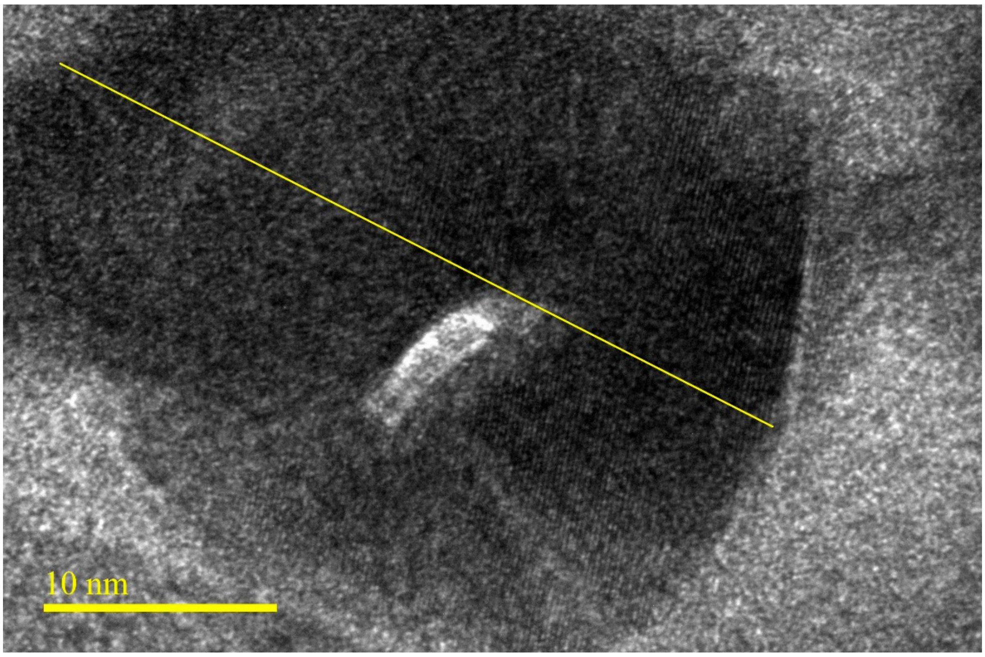

b)

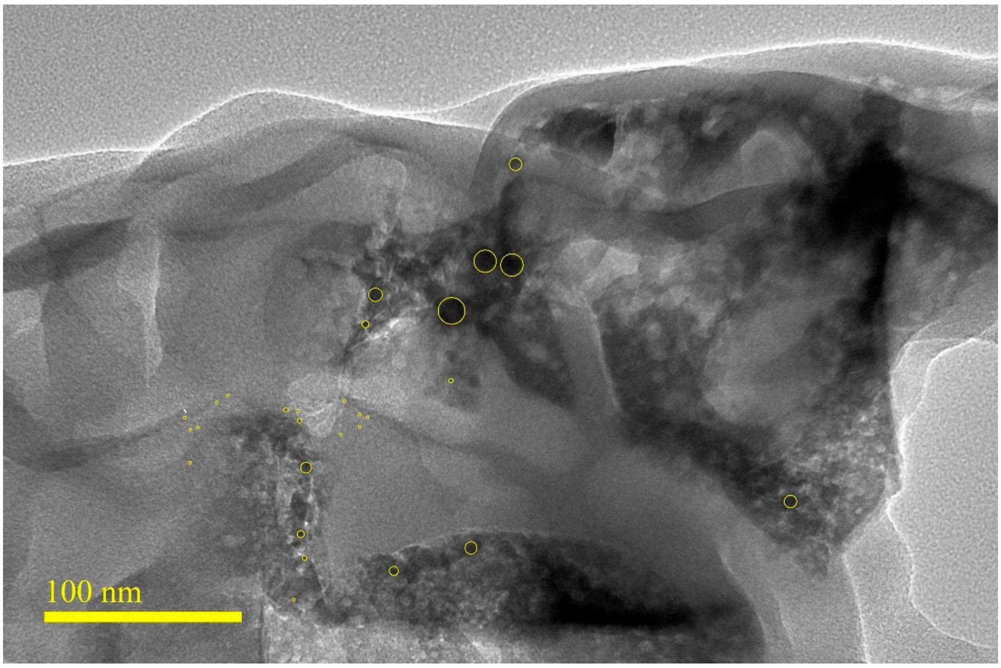

Figure S2: (a) HRTEM image of one copper particle (b) HRTEM showing several nanoparticles

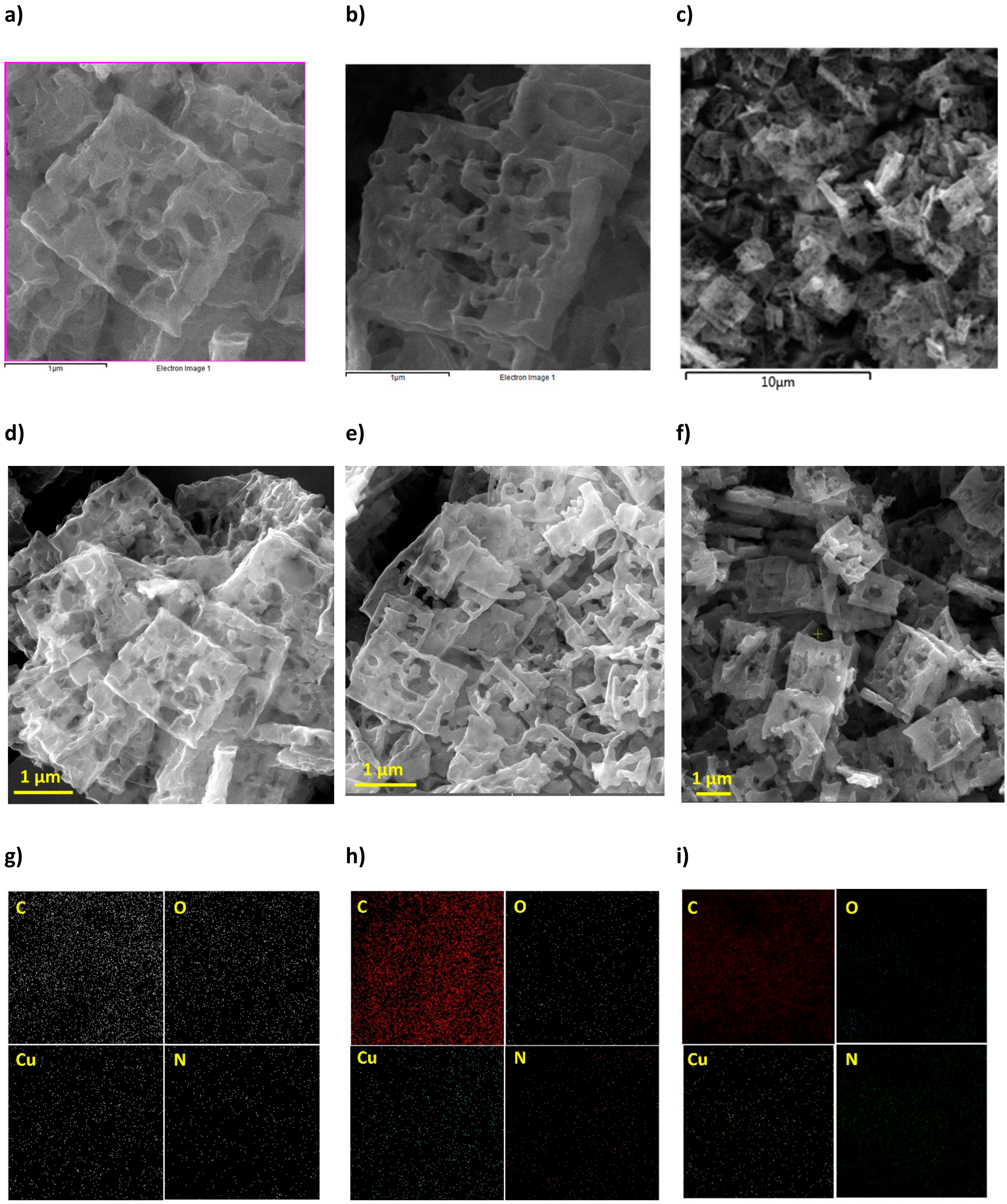

Figure S3: (a,d) SEM images of Cu/Cu_xO-PCC-0h (b,e) SEM images of Cu/Cu_xO-PCC-1h (c,f) SEM images of Cu/Cu_xO-PCC-6h (g,h,i) elemental mapping images for Cu/Cu_xO-PCC-0h, Cu/Cu_xO-PCC-1h, Cu/Cu_xO-PCC-6h, respectively

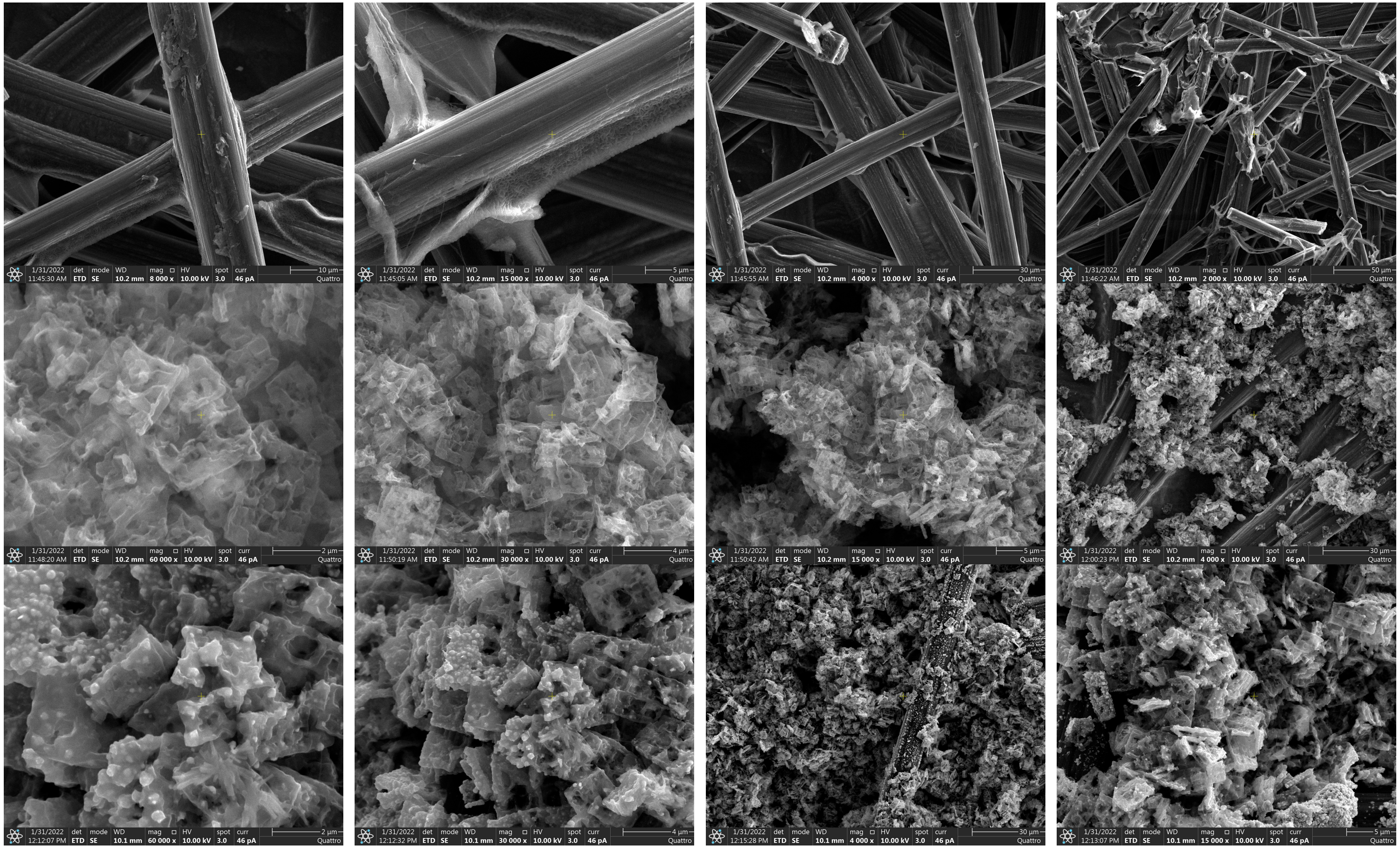

Figure S4: SEM images for the carbon paper (first row), Cu/CuxO-PCC-0h loaded in carbon paper before electrolysis (second row), after electrolysis (third row)

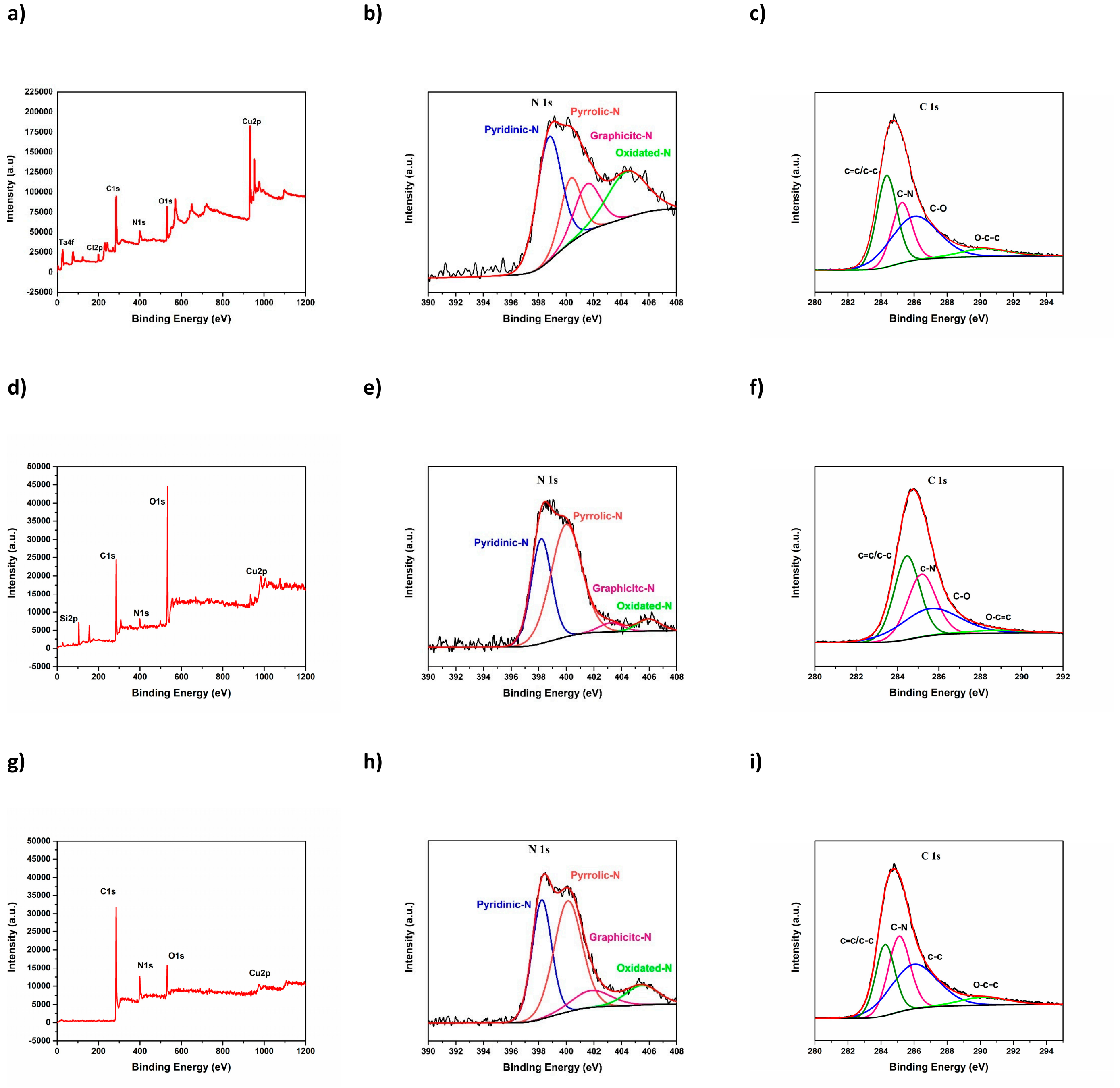

Figure S5: (a,d,g) XPS survey scans of Cu/Cu$_x$O-PCC-0h, Cu/Cu$_x$O-PCC-1h, Cu/Cu$_x$O-PCC-6h, respectively (b,e,h) XPS high resolution spectra of N 1s of Cu/Cu$_x$O-PCC-0h, Cu/Cu$_x$O-PCC-1h, Cu/Cu$_x$O-PCC-6h, respectively (c,f,i) XPS high resolution spectra of C 1s for of Cu/Cu$_x$O-PCC-0h, Cu/Cu$_x$O-PCC-1h, Cu/Cu$_x$O-PCC-6h, respectively

Table S1: current densities at different potentials

Potential vs RHE	Current density of Cu/Cu_xO-PCC-0h [mA/cm^2]	Current density of Cu/Cu_xO-PCC-1h [mA/cm^2]	Current density of Cu/Cu_xO-PCC-6h [mA/cm^2]
-0.5	0.119	0.0752	0.0627
-0.7	1.26	0.317	0.106
-0.9	2.00	0.750	0.566
-1.1	6.35	4.96	2.85

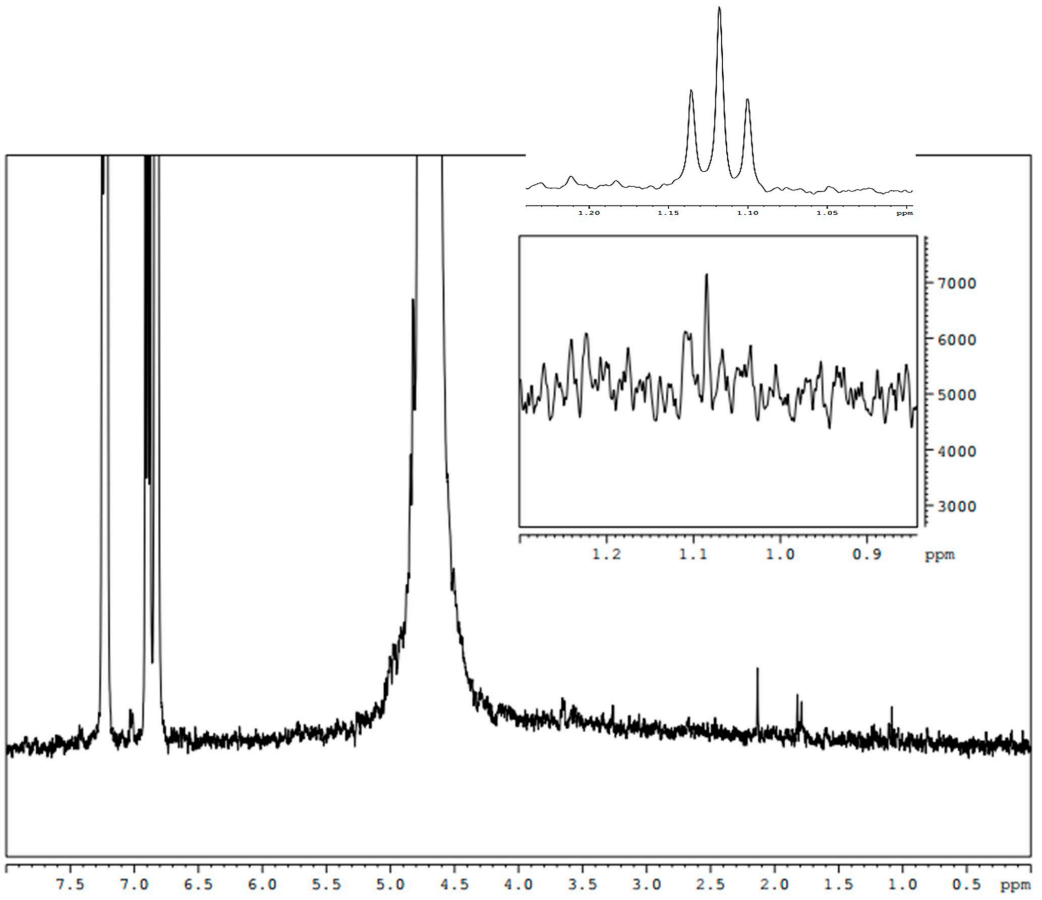

Figure S6: NMR spectrum showing Ethanol produced at -0.5 V vs RHE, ethanol peak from ethanol solution is shown in the top inset

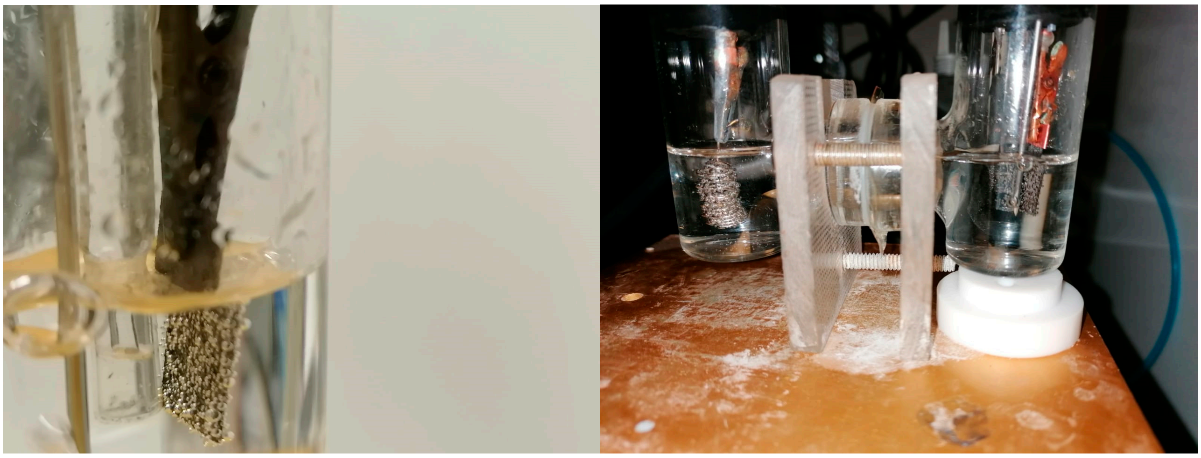

Figure S7: Electrochemical Cell and working electrode during electrolysis

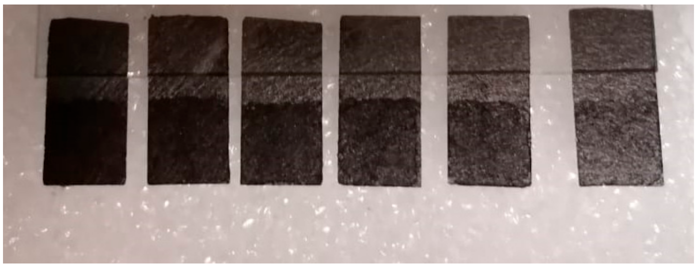

Figure S8: Working electrode
